# Rare association between cystic fibrosis, Chiari I malformation, and hydrocephalus in a baby: a case report and review of the literature

**DOI:** 10.1186/1752-1947-5-366

**Published:** 2011-08-12

**Authors:** Akash J Patel, Viraj H Raol, Andrew Jea

**Affiliations:** 1 Division of Pediatric Neurosurgery, Texas Children's Hospital, Department of Neurosurgery, Baylor College of Medicine, Houston, TX, USA

## Abstract

**Introduction:**

Cystic fibrosis, an epithelial cell transport disorder caused by mutations of the cystic fibrosis transmembrane conductance regulator gene, is not generally associated with malformations of the central nervous system. We review eight previously published reports detailing an infrequent association between cystic fibrosis and Chiari I malformation.

**Case presentation:**

To the best of our knowledge, our report describes only the ninth case of a baby presenting with a new diagnosis of cystic fibrosis and Chiari I malformation, in this case in a 10-month-old, full-term Caucasian baby boy from the United States of America. Neurosurgical consultation was obtained for associated developmental delay, macrocephaly, bulging anterior fontanel, and papilledema. An MRI scan demonstrated an extensive Chiari I malformation with effacement of the fourth ventricle, obliteration of the outlets of the fourth ventricle and triventricular hydrocephalus without aqueductal stenosis. Our patient was taken to the operating room for ventriculoperitoneal shunt placement.

**Conclusions:**

It is possible that the cystic fibrosis transmembrane conductance regulator gene may play a previously unrecognized role in central nervous system development; alternatively, this central nervous system abnormality may have been acquired due to constant valsalva from recurrent coughing or wheezing or metabolic and electrolyte imbalances that occur characteristically in cystic fibrosis.

## Introduction

Cystic fibrosis (CF) is a disorder in which transepithelial ion transport affects fluid secretion in exocrine glands and the epithelium of the respiratory, gastrointestinal, and reproductive tracts [1]. Although developmental abnormalities of the male genital tract are commonly associated with CF, malformations of other organ systems, particularly the central nervous system (CNS), are rarely associated with the disorder.

Chiari I malformation, defined by herniation of the cerebellar tonsils below the foramen magnum, is associated with hydrocephalus in 7% to 10% of cases [2,3]. Hydrocephalus often originates in the fourth ventricular outflow obstruction. An association between Chiari I malformation and cystic fibrosis has only been reported in the literature three times[4-6]. In the eight previously-reported patients with Chiari malformation, six patients had a known CF diagnosis, and two had an autopsy CF diagnosis. Here, we add the case of a ninth patient to the literature; a 10-month-old baby with a diagnosis of CF and associated Chiari I malformation and hydrocephalus. We also discuss the unusual association of these conditions.

## Case presentation

A 10-month-old, full-term Caucasian baby boy from the United States of America with a history of multiple respiratory infections, persistent cough, and greasy stools since birth, presented to our facility with failure to thrive and a one-week history of emesis. Our patient had a positive sweat chloride test result, confirming a new diagnosis of CF. Upon physical examination, we discovered he had macrocephaly with a head circumference in the 98th percentile, splaying of the sutures, and a bulging fontanel. A fundoscopic examination demonstrated papilledema. The remainder of his physical examination was unremarkable.

**Figure 1:**
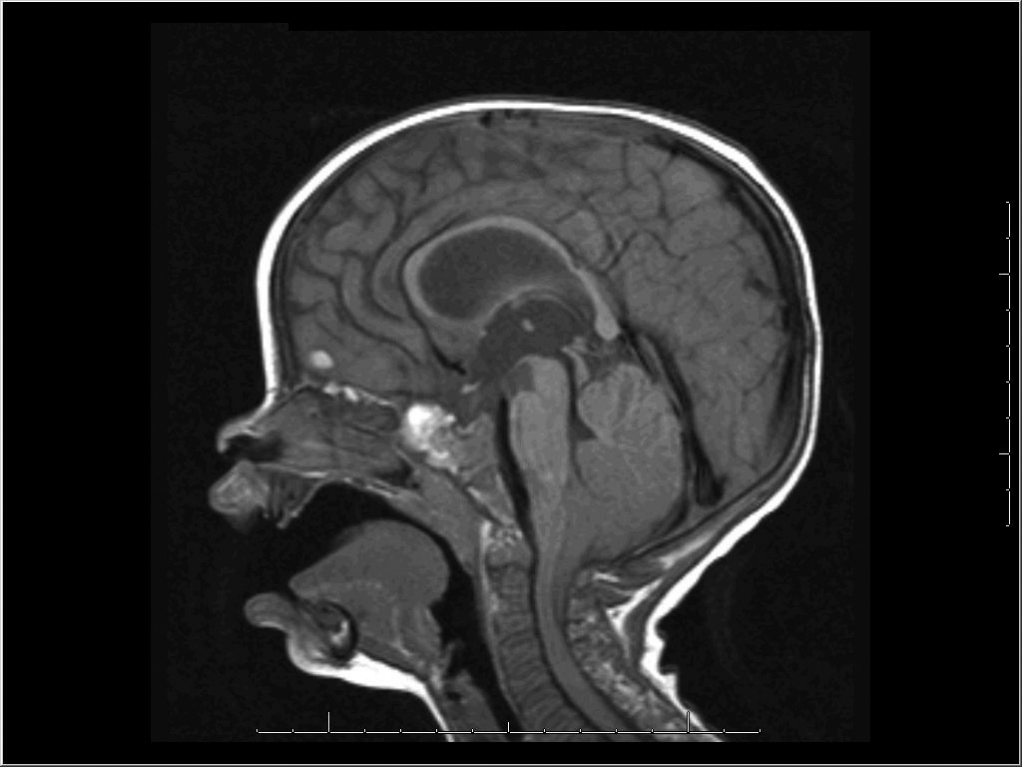
**Mid-sagittal T1 weighted MRI of the brain shows tonsillar herniation to the level of C3**. There is effacement of the fourth ventricle and Triventricular hydrocephalus.

Imaging studies demonstrated Triventricular hydrocephalus and severe Chiari I malformation with 2.2cm of cerebellar tonsillar herniation to the C3 level (Figure 1). Our patient did not have Chiari malformation symptoms at this time and was without signs of spinal cord compression or cerebellar or brainstem dysfunction. Therefore, we elected to treat the hydrocephalus first and followed our patient closely for the development of a problematic Chiari malformation.

Our patient was brought into the operating room for ventriculoperitoneal shunt (VPS) placement. The opening pressure was noted to be approximately 25 cm H_2_O upon cannulation of the ventricular system. The rest of the operation proceeded in standard fashion.

Our patient did well after surgery; he had no continued signs and symptoms of increased intracranial pressure and was discharged home the following day. At one-year follow-up with the Cystic Fibrosis Clinic and the Neurosurgery Service, our patient was continuing to do well with no signs or symptoms of increased intracranial pressure or problematic Chiari malformation.

## Discussion

### CF and Chiari I malformation

CF is an autosomal recessive disorder characterized by abnormal chloride conduction across epithelial membranes. It is the most common lethal genetic disease in the Caucasian population, with an incidence of one in 1500 to one in 4000 live births [1].

An association between CF and Chiari malformation has been previously reported in eight patients (Table 1) [4-6]. Rusakow *et al*. described a seven-year-old Hispanic girl with cystic fibrosis from homozygous delta F508 mutations who had cervicothoracic syringomyelia and a Chiari I malformation [6].

**Table 1:** Previous reports of cystic fibrosis (CF) and Chiari malformation

Reference	Age	Sex	Presenting signs and symptoms	Radiologic findings (extent of Chiari, ± aqueductal stenosis, ± hydrocephalus)	Treatment	Follow-up	Outcome
[10]	10 months	M	Swallowing dysfunction	Not reported	Chiari decompression	Not reported	Resolution of symptoms

[10]	18 years	M	Syncope	Not reported	Chiari decompression	Not reported	Resolution of symptoms

[10]	17 years	F	Numbness of extremities	Not reported	Chiari decompression	Not reported	Resolution of symptoms

[10]	three years	M	Recurrent vomiting	Not reported	None	Not reported	Symptoms unresolved

[10]	17 years	M	Persistent headache	Not reported	Chiari decompression	Not reported	Resolution of symptoms

[13]	four months	M	Failure to thrive	Aqueductal stenosis; hydrocephalus	VP shunt	three months	Death from sepsis

[13]	eight months	F	Failure to thrive, nystagmus	Tonsillar herniation with hydrocephalus	Chiari decompression and VP shunt	one year	Shunt malfunction and cerebral herniation

[15]	eight years	F	Thoracic scoliosis; gait instability; hand wasting	Chiari I with thoracic scoliosis and cervicothoracic syrinx	Chiari decompression	two months	Resolution of symptoms

Present report	10 months	M	Vomiting	2.2 cm of herniation; aqueductal stenosis; hydrocephalus	VP shunt	one month	Resolution of symptoms

VP = ventriculoperitoneal.

Needleman *et al*. described five patients who were known to have CF and were diagnosed with Chiari malformation after developing various neurological symptoms [4]. There were four males and one female ranging from 10 months to 18 years old. One of the patients was homozygous for the delta F508 mutation, while the others were heterozygous. In two patients, the second mutations could not be identified, while the remaining patients showed G542X and W1282X as their second mutation, respectively. The authors concluded that when patients with cystic fibrosis have unexplained neurological issues, Chiari I malformation is likely.

Rakheja *et al*. described two babies with a known diagnosis of Chiari I malformation and hydrocephalus; however, the CF diagnosis was not made until after death [5]. At presentation, the two patients, one boy and one girl, were four months and eight months old, respectively. One patient underwent Chiari decompression followed by shunt placement; the other underwent VPS placement as the initial treatment. The four-month-old boy died a month after surgery from acute bronchopneumonia leading to *Pseudomonas aeruginosa *sepsis. The eight-month-old girl died a year after surgery from increased intracranial pressure and possible shunt obstruction.

The mechanism of an association between cystic fibrosis and Chiari I malformation is unknown. It does not appear that the association is due to a specific type of cystic fibrosis transmembrane conductance regulator (CFTR) mutation as several different point mutations have been described in patients with both conditions [5]. It is possible that the CFTR gene plays an unrecognized role in CNS development. CFTR mRNA and protein expression have been identified in the developing human hypothalamus where the protein may be involved in neuropeptide vesicle trafficking [7,8]. Children with newly-diagnosed CF have transiently-elevated intracranial pressure which is thought to be due to either vitamin A deficiency, a response to treatment of their malnutrition [9], or intermittent valsalva from persistent, chronic wheezing or coughing [10,11]. Vitamin A also affects the development of this CNS/skeletal abnormality, as it induces abnormal cranial vault development and herniation of the cerebellum in hamster embryos [12].

### Chiari I malformation and hydrocephalus

In 1891, Hans Chiari was the first to describe the cerebellar tonsils descending below the foramen magnum [13]. However, the origin and pathophysiology of this condition remains poorly understood. Generally considered a congenital anomaly, some reports suggest that Chiari I malformation may be a sequelae of intracranial hypertension [14]. In his 1891 monograph, Chiari originally surmised that hydrocephalus pushed the cerebellar tonsils into the foramen magnum. But overt hydrocephalus is only reported in 7% to 10% of patients with symptomatic Chiari I malformation [15]. However, others have asserted that Chiari I malformation is a developmental anomaly of the posterior fossa with subsequent arachnoid adhesions, thought to be due to repeated trauma [16]. Therefore, any associated hydrocephalus may be due to fourth ventricular outflow obstruction [17]. It is possible that persistent coughing in CF could lead to arachnoid adhesions and altered CSF dynamics, resulting in hydrocephalus. This remains to be studied.

Krayenbuhl reported that 20 of 22 patients with symptomatic Chiari I malformation who were initially treated with ventriculoatrial shunt placement experienced symptom improvement [16]. Endoscopic third ventriculostomy may be an alternative to ventricular shunt placement for hydrocephalus associated with Chiari I malformation [15].

## Conclusions

In summary, we describe the case of a 10-month-old baby boy with a new diagnosis of CF and, later, Chiari malformation and hydrocephalus after presenting with developmental delay, macrocephaly, bulging anterior fontanel, and papilledema. We hypothesize that the CF precipitated the Chiari malformation although the mechanism is unclear. The extensive Chiari malformation then caused obstruction at the fourth ventricle outlets and resulted in hydrocephalus. Because of the young age of our patient, we elected to treat him with a VPS. His long-term clinical outcome remains to be determined.

## Consent

Written informed consent was obtained from the patient's next-of-kin for publication of this case report and any accompanying images. A copy of the written consent is available for review by the Editor-in-Chief of this journal.

## Competing interests

The authors declare that they have no competing interests.

## Authors' contributions

AJP and VHR were major contributors in the writing and editing of the manuscript. AJ was a major contributor in the interpretation and analysis of the data from our patient, and contributed to the design, writing, and editing of the manuscript. All authors read and approved the final manuscript.
